# Correction: TTC26/DYF13 is an intraflagellar transport protein required for transport of motility-related proteins into flagella

**DOI:** 10.7554/eLife.02897

**Published:** 2014-04-01

**Authors:** Hiroaki Ishikawa, Takahiro Ide, Toshiki Yagi, Xue Jiang, Masafumi Hirono, Hiroyuki Sasaki, Haruaki Yanagisawa, Kimberly A Wemmer, Didier YR Stainier, Hongmin Qin, Ritsu Kamiya, Wallace F Marshall

Ishikawa H, Ide T, Yagi T, Jiang X, Hirono M, Sasaki H, Yanagisawa H, Wemmer KA, Stainier DYR, Qin H, Kamiya R, Marshall WF. 2014. TTC26/DYF13 is an intraflagellar transport protein required for transport of motility-related proteins into flagella. *eLife* **3**:e01566. doi: http://dx.doi.org/10.7554/eLife.01566. Published 4 March 2014

In the published article, the legends for Videos 5 and 6 were switched.

This has been corrected.

